# Landmark clinical trials shaping contemporary guideline-directed medical therapy for heart failure

**DOI:** 10.1093/eschf/xvag141

**Published:** 2026-05-22

**Authors:** Michał Tkaczyszyn, Agnieszka Kapłon-Cieślicka, Maciej Janiszewski, Dominika Klimczak-Tomaniak, Ewa Jędrzejczyk-Patej, Paweł Burchardt, Amr Abdin, Michael Böhm, Ovidiu Chioncel, Guillaume Baudry, Marta Cobo Marcos, Robert Zymliński, Julio Núñez, Marco Metra, Piotr Ponikowski

**Affiliations:** Department of Cardiology, Clinical Department of Intensive Cardiac Care, Faculty of Medicine, Institute of Heart Diseases, Wroclaw Medical University, Borowska 213, Wroclaw 50-556, Poland; Department of Cardiology, Jan Mikulicz Radecki University Hospital in Wrocław, Borowska 213, Wroclaw 50-556, Poland; First Chair and Department of Cardiology, Medical University of Warsaw, Warsaw, Poland; Department of Heart Failure and Cardiac Rehabilitation, Medical University of Warsaw, Kondratowicza 8, Warsaw 03-242, Poland; Department of Heart Failure and Cardiac Rehabilitation, Medical University of Warsaw, Kondratowicza 8, Warsaw 03-242, Poland; Department of Cardiology, Hypertension and Internal Medicine, Mazovian Bródno Hospital, Kondratowicza 8, Warsaw 03-242, Poland; First Department of Cardiology and Angiology, Silesian Centre for Heart Diseases, Zabrze, Poland; Department of Cardiology, J. Strus Hospital, Poznan, Poland; Department of Hypertension, Angiology, and Internal Medicine, Poznan University of Medical Sciences, Poznan, Poland; Department of Internal Medicine III, Cardiology, Angiology, Intensive Care Medicine, Saarland University Medical Center, Saarland University, Homburg/Saar, Germany; HOMICAREM (HOMburg Institute for CArdioREnalMetabolic Medicine), Faculty of Medicine, Campus Universitätsklinikum des Saarlandes, Saarland University, Homburg, Germany; Emergency Institute for Cardiovascular Diseases ‘Prof. C. C. Iliescu’, University of Medicine ‘Carol Davila’, Bucharest, Romania; Université de Lorraine, INSERM, Centre D'Investigation Clinique Plurithématique 1433, Inserm U1116, CHRU de Nancy, Nancy, France; INI-CRCT (Cardiovascular and Renal Clinical Trialists) F-CRIN Network, Nancy, France; REICATRA, Recherche et Enseignement en IC Avancée, Transplantation, Assistance, Vandœuvre-lès-Nancy, France; Cardiology Department, Hospital Universitario Puerta de Hierro, C/Joaquín Rodrigo 2, Madrid 28222, Spain; Centro de Investigación Biomédica en Red (CIBER) Cardiovascular, C/Monforte de Lemos 3-5, Pabellón 11, Madrid 28029, Spain; Department of Cardiology, Clinical Department of Intensive Cardiac Care, Faculty of Medicine, Institute of Heart Diseases, Wroclaw Medical University, Borowska 213, Wroclaw 50-556, Poland; Department of Cardiology, Jan Mikulicz Radecki University Hospital in Wrocław, Borowska 213, Wroclaw 50-556, Poland; Department of Cardiology, Hospital Clínico Universitario de Valencia, Valencia, Spain; Department of Medicine, University of Valencia, Valencia, Spain; Institute of Cardiology, ASST Spedali Civili, Department of Medical and Surgical Specialties, Radiologic Sciences and Public Health, University of Brescia, Brescia, Italy; Department of Cardiology, Clinical Department of Intensive Cardiac Care, Faculty of Medicine, Institute of Heart Diseases, Wroclaw Medical University, Borowska 213, Wroclaw 50-556, Poland; Department of Cardiology, Jan Mikulicz Radecki University Hospital in Wrocław, Borowska 213, Wroclaw 50-556, Poland

**Keywords:** Guideline-directed medical therapy, Pharmacotherapy, Heart failure

## Abstract

Heart failure (HF) management has evolved substantially over recent decades, transitioning from predominantly symptomatic treatment to a strategy grounded in disease-modifying therapies that improve survival and reduce hospitalizations. This review provides a structured and clinically oriented synthesis of landmark randomized controlled trials (RCTs) that have shaped contemporary guideline-directed medical therapy across the spectrum of left ventricular ejection fraction (LVEF). Evidence from pivotal large-scale RCTs is presented according to pharmacological classes, encompassing both foundational and selected adjunctive therapies. In HF with reduced LVEF, multiple drug classes consistently reduce mortality and HF hospitalizations, forming the basis of contemporary quadruple therapy. In contrast, evidence in HF with mildly reduced or preserved LVEF remains more heterogeneous, with sodium–glucose co-transporter-2 inhibitors emerging as the only class with consistent benefit across the full LVEF spectrum. Additional therapies, including digoxin, vericiguat, intravenous iron, and emerging incretin-based agents, provide incremental benefits in selected clinical contexts. Differences in trial populations, design, and background therapy limit direct comparisons but highlight the progressive refinement of HF treatment strategies. Despite the strength of the evidence base, a persistent gap remains between guideline recommendations and real-world implementation. Bridging this gap, improving representation of under-represented populations in clinical trials, and advancing precision medicine approaches remain key priorities for future research.

## Introduction

**Figure 1:**
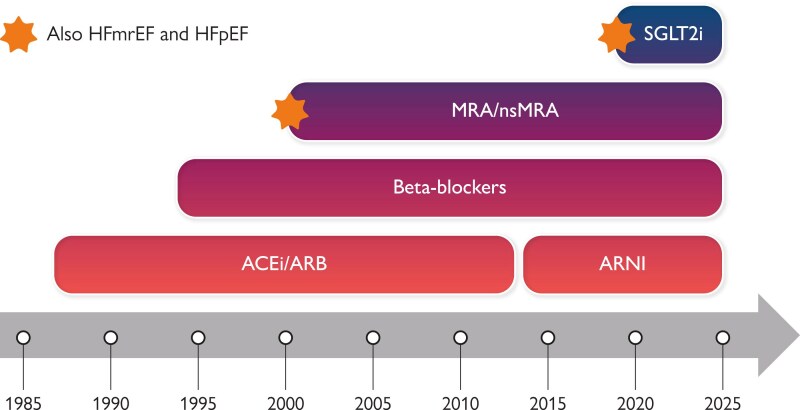
Timeline of the introduction of major drug classes improving outcomes in heart failure with reduced ejection fraction. Abbreviations: ACEI, angiotensin-converting enzyme inhibitors; ARB, angiotensin receptor blockers; ARNI, angiotensin receptor–neprilysin inhibitors; MRA, mineralocorticoid receptor antagonists; nsMRA, non–steroidal mineralocorticoid receptor antagonists; SGLT2i, sodium–glucose co–transporter 2 inhibitors; HFmrEF, heart failure with mildly reduced ejection fraction; HFpEF, heart failure with preserved ejection fraction

**Figure 2:**
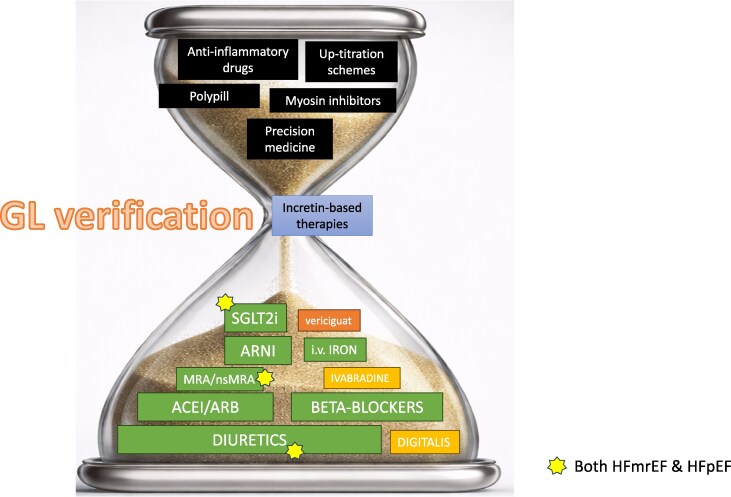
Evolution of pharmacotherapy composition and strategy in heart failure with reduced ejection fraction: established therapies and agents under evaluation - an illustrative figure. Abbreviations: ACEI, angiotensin-converting enzyme inhibitors; ARB, angiotensin receptor blockers; ARNI, angiotensin receptor-neprilysin inhibitors; MRA, mineralocorticoid receptor antagonists; nsMRA, non-steroidal mineralocorticoid receptor antagonists; SGLT2i, sodium-glucose co-transporter 2 inhibitors; HFmrEF, heart failure with mildly reduced ejection fraction; HFpEF, heart failure with preserved ejection fraction

Heart failure (HF) therapy has changed dramatically over the past decades. A field once dominated by symptomatic treatment has evolved into one grounded in therapies proven to improve quality of life and reduce morbidity and mortality. This progress has been driven by landmark randomized controlled trials (RCTs), which have shaped contemporary guideline-directed medical therapy (GDMT) across the spectrum of left ventricular ejection fraction (LVEF) (*Figure 1 and Figure 2*). The purpose of this document is to bring together, in a single accessible and clinically oriented review, the key large RCTs that established specific pharmacological therapies as treatments recommended or considered to improve outcomes in HF. Trial selection included studies that substantially influenced international guideline recommendations or clinical practice.

To simplify the message, we present the evidence by drug class and, within each class, summarize the major trial data for HF with reduced LVEF (HFrEF) and, where available, HF with preserved LVEF (HFpEF). We consider therapies evaluated in patients with LVEF <50% as part of the broader reduced ejection fraction spectrum, acknowledging that contemporary guidelines distinguish HFrEF (≤40%) from HFmrEF (41%–49%). Importantly, HF trial populations have evolved substantially over time, with clear differences in patient characteristics, background therapy, trial design, and LVEF thresholds. These differences limit direct comparisons, but they also illustrate the remarkable therapeutic journey of HF management. By presenting these selected landmark studies in a single coherent document, we aim to demonstrate how decades of clinical investigation have shaped the current understanding and treatment of HF.

### Diuretics

Diuretics were first introduced into HF management in the 1960s, and their clinical use expanded over the following years.^1,2^ Loop diuretics, particularly furosemide, became a cornerstone of HF care because they provided rapid and often dramatic relief of dyspnoea and peripheral congestion.^3,4^ Their adoption was therefore driven primarily by clear clinical efficacy rather than by large RCTs demonstrating reductions in mortality or other hard outcomes. As emphasized in contemporary guidelines, diuretics remain the cornerstone for alleviating congestion and symptom improvement, but they are not classic disease-modifying, mortality-reducing drugs mainly due to lack of well-powered RCTs.^5–10^ Although some studies demonstrate an association between diuretic use and increased mortality in HF, not all observations confirm this finding; moreover, this relationship may reflect more advanced disease severity, necessitating higher drug doses to maintain euvolemia, and no causality can be claimed.^11–17^ They may have untoward effects on the clinical course of HF, causing neurohormonal activation, electrolyte disturbances, and possibly kidney dysfunction. However, consistently their dosing and administration are currently diminishing with the adoption of GDMT.^18–20^

### Angiotensin-converting enzyme inhibitors

**Figure 3:**
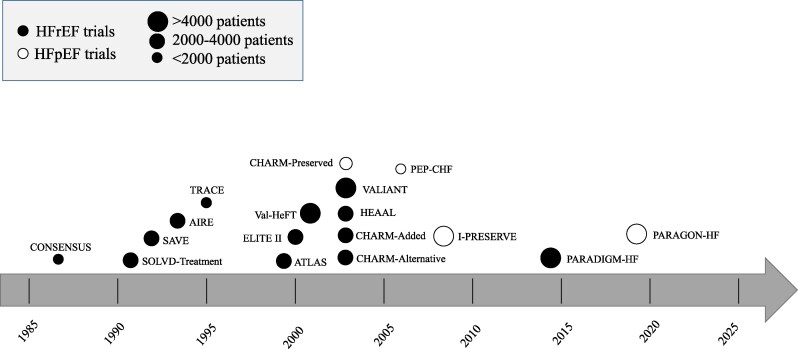
Timeline of major clinical trials evaluating multi-level renin-angiotensin-aldosterone system blockade in heart failure, with study size and heart failure phenotype indicated. Abbreviations: HFrEF, heart failure with reduced ejection fraction; HFpEF, heart failure with preserved ejection fraction

Angiotensin-converting enzyme inhibitors (ACEi) were introduced into clinical practice in the 1980s, initially as antihypertensive agents. Advances in understanding HF pathophysiology—particularly the central role of sustained activation of the renin–angiotensin–aldosterone system (RAAS)—led to its evaluation as a disease-modifying therapy in HF (*Table 1* and *Figure 3*).

**Table 1:** Clinical trials targeting renin–angiotensin–aldosterone system in heart failure

Trial (year of publication)	Intervention	Number of patients	Special information about the population	Primary outcome	Events: intervention vs comparator	Effect measure
ACEI						
CONSENSUS (1987)	Enalapril vs placebo	253	Severe symptomatic HF (NYHA IV)	All-cause mortality (6 months)	26% vs 44%	Risk reduction 40%; *P* = .002
SOLVD-Treatment (1991)	Enalapril vs placebo	2569	Symptomatic HF, LVEF ≤35% (NYHA II–III)	All-cause mortality	35% vs 40%	RR: .84 (95% CI .74–.95); *P* = .004
SAVE (1992)	Captopril vs placebo	2231	Post-MI (3–16 days), LVEF ≤40%	All-cause mortality	20% vs 25%	RR: .81 (95% CI .68–.97); *P* = .019
AIRE (1993)	Ramipril vs placebo	2006	Post-MI with clinical HF	All-cause mortality	17% vs 23%	RR: .73 (95% CI .60–.88); *P* = .002
TRACE (1995)	Trandolapril vs placebo	1749	Post-MI, LVEF ≤35%	All-cause mortality	35% vs 42%	RR: .78 (95% CI .67–.91); *P* = .001
ATLAS (1999)	Lisinopril high-dose vs low-dose	3164	Chronic symptomatic HF (NYHA II–IV), LVEF ≤30%	All-cause mortality	20% vs 21%	HR: .92 (95% CI .82–1.03); *P* = .128
PEP-CHF (2006)	Perindopril vs placebo	850	HF with EF >40%; age ≥70 years; symptomatic HF (mainly NYHA II–III); evidence of diastolic dysfunction	All-cause mortality or HF hospitalization	24% vs 25%	HR: .92 (95% CI .70–1.21); *P* = .545
ARB						
ELITE II (2000)	Losartan vs captopril	3152	≥60 years, NYHA II–IV, LVEF ≤40%	All-cause mortality	18% vs 16% (annual mortality 12% vs 10%)	HR: 1.13 (95% CI .95–1.35); *P* = .16
Val-HeFT (2001)	Valsartan vs placebo (background therapy)	5010	NYHA II–IV; ARB added to standard therapy	Composite: all-cause mortality + morbidity	723 (29%) vs 801 (32%)	RR: .87 (95% CI .77–.97); *P* = .009
CHARM-Alternative (2003)	Candesartan vs placebo	2028	Symptomatic HF, LVEF ≤40%; ACE-I intolerant	CV death or HF hospitalization	334 (33%) vs 406 (40%)	HR: .77 (95% CI .67–.89); *P* < .001
CHARM-Added (2003)	Candesartan + ACE-I vs placebo + ACE-I	2548	NYHA II–IV, LVEF ≤40%; all on ACE-I (55% β-blocker, 17% MRA)	CV death or HF hospitalization	483 (38%) vs 538 (42%)	HR: .85 (95% CI .75–.96); *P* = .011
HEAAL (2009)	Losartan 150 mg vs 50 mg	3846	HFrEF (LVEF ≤40%), NYHA II–IV; ACE-I intolerant	All-cause death or HF hospitalization	828 (43%) vs 889 (46%)	HR: .90 (95% CI .82–.99); *P* = .027
VALIANT (2003)	Valsartan vs captopril (post-MI)	14 703	5–10 days post-MI with HF and/or LV dysfunction	All-cause mortality	979 (20%) vs 958 (20%)	HR: 1.00 (95% CI .90–1.11); *P* = .98
CHARM-Preserved (2003)	Candesartan vs placebo	3023	Symptomatic HF (NYHA II–IV), LVEF >40%	CV death or HF hospitalization	333 (22%) vs 366 (24%)	HR: .89 (95% CI .77–1.03); *P* = .118
I-PRESERVE (2008)	Irbesartan vs placebo	4128	HFpEF (LVEF ≥45%)	All-cause death or CV hospitalization	742 (36%) vs 763 (37%)	HR: .95 (95% CI .86–1.05); *P* = .35
ARNI						
PARADIGM-HF (2014)	Sacubitril/valsartan vs enalapril	8442	Chronic symptomatic HFrEF on background guideline-directed therapy	CV death or first HF hospitalization	22% vs 27%	HR: .80 (95% CI .73–.87); *P* < .001
PARAGON-HF (2019)	Sacubitril/valsartan vs valsartan	4822	Symptomatic HFpEF (LVEF ≥45%); recurrent-event analysis	Total HF hospitalizations and CV death	894 total events in 526 patients vs 1009 total events in 557 patients	Rate ratio: .87 (95% CI .75–1.01); *P* = .06

If not otherwise specified, events and effect estimates refer to the primary endpoint.

The first unequivocal demonstration of survival benefit was provided by the CONSENSUS trial, which established ACE inhibition as life-prolonging therapy in patients with advanced symptomatic HFrEF. These findings were subsequently extended to a broader population with milder symptomatic disease in the SOLVD-treatment trial, firmly establishing ACEi as foundational therapy in HFrEF.^21,22^ The SAVE study demonstrated that early initiation of captopril in patients with left ventricular dysfunction after myocardial infarction (MI) reduced mortality and delayed the development of overt HF. Similarly, the AIRE trial confirmed the benefit of early ramipril therapy in patients with clinical HF following acute MI. The TRACE study further expanded the evidence to patients with left ventricular dysfunction after MI, including those without overt HF symptoms, reinforcing the concept that RAAS inhibition modifies disease trajectory even at earlier stages of ventricular dysfunction.^23–25^ Once the survival benefit of ACE inhibitors had been firmly established, attention shifted to dose optimization. The ATLAS trial evaluated the clinical impact of higher vs lower doses of lisinopril. Although higher doses did not significantly reduce all-cause mortality, they were associated with fewer HF-related events, supporting titration to maximally tolerated target doses used in clinical trials.^26^

Collectively, these landmark studies demonstrated that ACE inhibitors reduce mortality, decrease hospitalization rates, and slow disease progression across the spectrum of HFrEF—from advanced symptomatic HF to asymptomatic left ventricular dysfunction following MI. In contrast, evidence supporting ACE inhibitor therapy in HF with preserved ejection fraction (HFpEF) remains limited. Although the PEP-CHF trial was underpowered due to lower-than-anticipated recruitment and event rates, it did not demonstrate a significant reduction in the primary composite endpoint in the overall population.^27^ Consequently, ACE inhibitors in HFpEF are primarily used for the treatment of comorbid conditions such as hypertension post-MI (AIRE) or coronary artery disease (HOPE, ONTARGET, PEACE, and EUROPA) rather than as disease-modifying therapy in HFpEF.^27^

### Angiotensin receptor blockers

Despite the theoretical advantages of angiotensin receptor blockers (ARBs), RCTs have not demonstrated superiority over ACEi in HFrEF. The ELITE II trial, comparing losartan with captopril in patients with symptomatic HFrEF, showed no reduction in all-cause mortality with losartan, although treatment was associated with improved tolerability. These findings established ARBs primarily as an alternative for patients unable to tolerate ACE inhibitors rather than as preferred first-line therapy.^28^ The Val-HeFT trial evaluated the addition of valsartan to background therapy (fixed-dose drug regimen that could include ACE inhibitors, diuretics, digoxin, and beta-blockers for ≥2 weeks). Although valsartan did not reduce all-cause mortality, it reduced a composite endpoint of mortality and morbidity, driven mainly by a reduction in HF hospitalizations. *Post-hoc* analyses suggested heterogeneity of treatment effects depending on background therapy, raising concerns regarding routine ACEi+ARB therapy.^29^

The CHARM programme further clarified the role of candesartan in HFrEF. In CHARM-alternative, which enrolled patients intolerant to ACE inhibitors, candesartan reduced the composite endpoint of cardiovascular death or HF hospitalization. In CHARM-added, candesartan provided incremental benefit when added to ACEi therapy, although this was accompanied by increased rates of hyperkalaemia and renal dysfunction, highlighting the need for careful monitoring when combining RAAS inhibitors.^30,31^

ARBs were also evaluated in patients with MI complicated by left ventricular dysfunction or HF. In the VALIANT trial, valsartan was non-inferior to captopril with respect to all-cause mortality, while combination therapy did not provide additional survival benefit and was associated with more adverse events. Similarly, the OPTIMAAL trial did not demonstrate superiority of losartan over captopril after acute MI. Together, these studies reinforced ACE inhibitors as standard therapy while supporting ARBs as suitable alternatives in cases of intolerance.^32,33^ Dose optimization was addressed in the HEAAL trial, which showed that higher-dose losartan reduced the composite of death or HF hospitalization compared with lower-dose therapy in patients intolerant to ACE inhibitors, supporting titration to maximally tolerated target doses.^34^

In HFpEF, the results of ARB trials have been less convincing. Both CHARM-Preserved and I-PRESERVE failed to demonstrate significant reductions in mortality in the overall populations studied. Although modest reductions in HF hospitalizations were observed in some analyses, consistent prognostic benefit has not been established.^35,36^

### Angiotensin receptor–neprilysin inhibitors

The PARADIGM-HF trial established sacubitril/valsartan as a transformative therapy in HFrEF. In this landmark study, sacubitril/valsartan was compared with enalapril in patients with symptomatic HFrEF receiving guideline-directed therapy, demonstrating significant reductions in cardiovascular death and HF hospitalization as well as improved overall survival, and fundamentally changing the therapeutic paradigm in HFrEF.^37^

Subsequent studies examined the timing of initiation of angiotensin receptor–neprilysin inhibitors (ARNIs) in clinical practice. In the PIONEER-HF trial, initiation of sacubitril/valsartan in hemodynamically stabilized patients hospitalized for acute decompensated HFrEF produced a greater reduction in NT-proBNP concentrations compared with enalapril, with exploratory analyses suggesting fewer HF rehospitalizations.^38^ The TRANSITION study further demonstrated the feasibility and safety of initiating sacubitril/valsartan either before hospital discharge or shortly thereafter, supporting early implementation in routine practice.^39^

ARNI therapy has also been investigated in HFpEF.^40,41^ In the PARAGON-HF trial, sacubitril/valsartan did not achieve a statistically significant reduction in the primary composite endpoint of total HF hospitalizations and cardiovascular death compared with valsartan alone in HF patients with NYHA II-IV, LVEF≥45%, elevated natriuretic peptides, and structural heart disease. Although the overall result was neutral, additional analyses of individual patient data from the PARAGON-HF and PARADIGM-HF trials suggest potential benefits for certain endpoints in patients with HF with mildly reduced LVEF.^42,43^

Overall, clinical trials over the past decade have established sacubitril/valsartan as a cornerstone therapy in HFrEF, where it improves morbidity and mortality and is recommended as first-line therapy in contemporary guidelines.^5,6,44^ In HFpEF, its role remains more limited, appearing confined to selected patient subgroups.

### Beta-adrenergic receptor antagonists (β-blockers)

**Figure 4:**
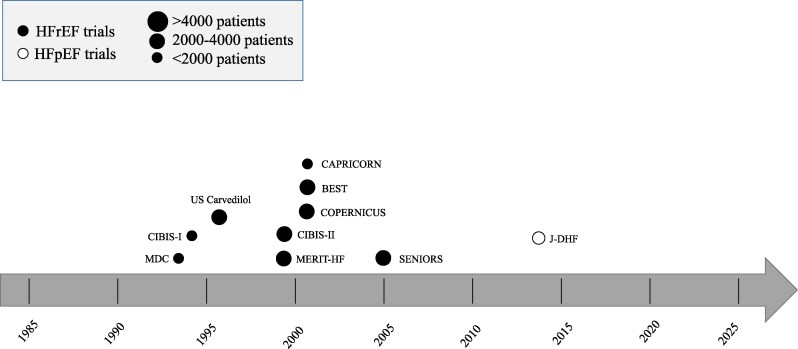
Timeline of major clinical trials evaluating beta-adrenergic system blockade in heart failure, with study size and heart failure phenotype indicated. Abbreviations: HFrEF, heart failure with reduced ejection fraction; HFpEF, heart failure with preserved ejection fraction

Until the late 1990s, β-blockers were contraindicated in HF. Studies in the late 1990s showed their efficacy in reducing morbidity and mortality. Key trials—such as MERIT-HF (metoprolol succinate), CIBIS-II (bisoprolol), COPERNICUS (carvedilol), and SENIORS (nebivolol)—demonstrated reductions in all-cause and cardiovascular mortality that shaped the guidelines (*Table 2* and *Figure 4*).^45–50^ No significant reduction in mortality was demonstrated for bucindolol.^51^ Meta-analyses confirm a 30%–35% relative reduction in all-cause mortality among patients with LVEF ≤40% treated with β-blockers (metoprolol, bisoprolol, carvedilol, and nebivolol).^52–55^ Importantly, benefit was observed across subgroups, including older patients, those with ischemic and non-ischemic aetiologies, and patients with mild to severe symptoms. Beta-blockers also significantly reduce the incidence of SCD.^53,56^ Thus, this group remains a foundational therapy and should be initiated early and uptitrated to the maximally tolerated dose alongside other pillars of HF therapy.

**Table 2:** Clinical trials supporting the use of β-blockers in heart failure

Trial (year of publication)	Intervention	Number of patients	Special information about the population	Primary outcome	Events: intervention vs comparator	Effect measure
HFrEF						
MDC (1993)	Metoprolol Tartrate vs placebo	383	LVEF <40%, dilated cardiomyopathy	All-cause mortality or progression to heart transplantation	25 vs 38	HR: .66 (95% CI .38–.94)
MERIT-HF (1999)	Metoprolol Succinate vs placebo	3991	LVEF ≤40%, NYHA II–IV	All-cause mortality	145 vs 217	HR: .66 (95% CI .53–.81)
CIBIS-I (1994)	Bisoprolol vs placebo	641	LVEF <40%, NYHA III-IV	All-cause mortality	53 vs 67	HR: .80 (95% CI .56–1.15)
CIBIS-II (1999)	Bisoprolol vs placebo	2647	LVEF ≤35%, NYHA III–IV	All-cause mortality	156 vs 228	HR: .66 (95% CI .54–.81)
US Carvedilol (1996)	Carvedilol vs placebo	1094	LVEF ≤35%, mild, moderate, or severe HF	All-cause mortality	22 vs 31	HR: .35 (95% CI .20–.61)
COPERNICUS (2001)	Carvedilol vs placebo	2289	LVEF <25%, severe HF	All-cause mortality	130 vs 190	HR: .65 (95% CI .52–.81)
BEST (2001)	Bucindolol vs placebo	2708	LVEF ≤35%, NYHA III-IV	All-cause mortality	411 vs 449	HR: .90 (95% CI .78–1.02)
SENIORS (2005)	Nebivolol vs placebo	2128	Age ≥70 years; LVEF ≤35% within previous 6 months or HFH within previous 1 y regardless of LVEF	Composite of all-cause death or CV hospitalization	332 vs 375	HR: .86 (95% CI .74–.99)
CAPRICORN (2001)	Carvedilol vs placebo	1959	Post-MI, LVEF ≤40%	All-cause mortality or CV hospitalization (secondary: all-cause mortality)	340 vs 367 (secondary: 116 vs 151)	HR: .92 (95% CI .80–1.07) [secondary: .77 (95% CI .60–.98)]
HFpEF						
J-DHF (2013)	Carvedilol vs placebo	245	LVEF ≥40% (majority ≥50%)	Composite of CV death or HFH	29 vs 34	HR: .90 (95% CI .55–1.49)

If not otherwise specified, events and effect estimates refer to the primary endpoint.

Randomized trials specifically targeting HFmrEF are scarce. Evidence mainly comes from subgroup analyses of HFrEF trials and patient-level meta-analyses.^57,58^ A meta-analysis of double-blind RCTs found that β-blockers reduce cardiovascular mortality in patients with sinus rhythm and LVEF 40%–49%, with diminishing benefit at higher EF levels (*Table 2*).^57^ This benefit appears weaker than in classic HFrEF and, as shown by large meta-analyses, may be influenced by rhythm status. β-Blocker therapy reduced mortality in patients with sinus rhythm but did not have the same effect in patients with atrial fibrillation, as indicated by a significant interaction *P*-value for baseline rhythm.^59^

In HFpEF, no large RCT has demonstrated a clear mortality benefit of β-blockers. Available data come from small RCTs, observational studies, and subgroup analyses of mixed-LVEF trials. In observational studies, β-blocker therapy was linked to reduced all-cause mortality but did not affect HF hospitalizations.^60^ Conversely, in RCTs such as J-DHF and ELANDD, β-blocker use was not associated with all-cause mortality and did not improve exercise capacity.^61,62^ Moreover, in HFpEF, heart rate reduction may impair exercise tolerance in patients with chronotropic incompetence, a common feature in this population. In a small RCT in symptomatic HFpEF with chronotropic incompetence, β-blocker withdrawal led to a significant short-term improvement in maximum functional capacity.^63^ Notably, β-blockers are often prescribed in HFpEF due to other indications like atrial fibrillation, ischemic heart disease, or hypertension. In these cases, their use is supported by comorbidities rather than by specific outcome data related to HFpEF. In summary, for HFrEF, β-blockers are a core part of GDMT with clear mortality benefits.^5^ In HFmrEF, the benefit appears reduced and depends on the rhythm, whereas in HFpEF, no clear prognostic advantage has been shown, except in certain comorbid conditions.

### Mineralocorticoid receptor antagonists

The ‘story’ of mineralocorticoid receptor antagonist (MRA) use in HFrEF began in 1999 with the RALES (spironolactone) trial.^64^ In the trial that recruited severe HFrEF (LVEF<35%, at NYHA III or IV at the time of enrolment who were treated with an ACEi and a loop diuretic), adding spironolactone to the therapy significantly reduced (by ∼30%) the risk of death from any cause (study primary outcome) as well as the risk of worsening of HF (by ∼35%).^64^ This landmark trial showed that MRA was not merely a symptomatic treatment but a therapy capable of improving prognosis. The benefits of MRA therapy were then extended to patients after acute MI with left ventricular dysfunction and clinical HF in the EPHESUS trial.^65^ In EPHESUS, eplerenone significantly reduced mortality and cardiovascular events (composite primary outcome), strengthening the role of this drug class in high-risk patients.^65^ Based on this, eplerenone was then tested in less symptomatic HFrEF patients (NYHA II, EF ≤ 35%) in the EMPHASIS-HF (eplerenone) trial.^66^ This trial was stopped prematurely, as it confirmed that eplerenone is effective in this HFrEF population as well. Eplerenone significantly reduced (∼37%) the risk of cardiovascular death or HF hospitalization (primary endpoint).^66^

**Figure 5:**
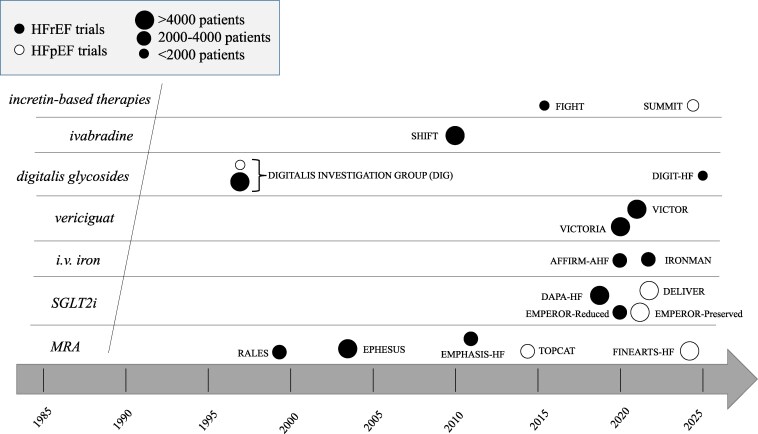
Timeline of major clinical trials evaluating other key therapies in heart failure, with study size and heart failure phenotype indicated. Abbreviations: HFrEF, heart failure with reduced ejection fraction; HFpEF, heart failure with preserved ejection fraction; i.v., intravenous; SGLT2i, sodium-glucose co-transporter type 2 inhibitor; MRA, mineralocorticoid receptor antagonists.

The next attempt to translate the benefits of MRAs into HFpEF produced mixed results. The TOPCAT trial tested the use of spironolactone in the HFpEF population and did not result in improvement of the study’s primary outcome (composite of cardiovascular death, aborted cardiac arrest, or HF hospitalizations), although there was a signal towards fewer HF hospitalizations.^67^ It should be acknowledged that significant geographical heterogeneity in study results was observed in the TOPCAT trial, and detailed *post-hoc* analyses are available elsewhere.^68^ In 2024, the FINEARTS-HF (finerenone) was introduced—the emergence of a non-steroidal MRA marked a new phase in this treatment approach. In patients with HFmrEF/HFpEF, finerenone lowered the risk of worsening HF events and cardiovascular death, providing some of the strongest evidence to date that MRA may also enhance outcomes beyond traditional HFrEF (*Table 3* and *Figure 5*).^69^ The results mentioned above were also validated in the meta-analysis.^70^

**Table 3:** Clinical trials supporting the use of mineralocorticoid receptor antagonists in heart failure

Trial (year of publication)	Intervention	Number of patients	Special information about the population	Primary outcome	Events: intervention vs comparator	Effect measure
HFrEF						
RALES (1999)	Spironolactone vs placebo	1663	Severe HF with LVEF≤35%	Death from all causes	284 (35%) vs 386 (46%)	RR: .70 (95% CI .60; .82); *P* < .001
EPHESUS (2003)	Eplerenone vs placebo	6632	Post-MI HF	Death from any cause and death from cardiovascular causes or hospitalization for heart failure, acute myocardial infarction, stroke, or ventricular arrhythmia.	478 vs 554 CV deaths: 407 vs 483	RR: .85 (95% CI .75–.96); *P* = .008 RR: .83 (95% CI .72–.94); *P* = .005
EMPHASIS-HF (2011)	Eplerenone vs placebo	2737	NYHA class II and LVEF≤35%	Composite of death from cardiovascular causes or hospitalization for heart failure.	18% vs 26%	HR .63 (95% CI .54–.74); *P* < .001
HFpEF						
TOPCAT (2014)	Spironolactone vs placebo	3445	Symptomatic HF with LVEF≥45%	Composite of CV death, aborted cardiac arrest, or HF hospitalization	320 (19%) vs 351 (20%)	HR: .89 (95% CI .77–1.04); *P* = .14
FINEARTS-HF (2024)	Finerenone vs placebo	6001	LVEF≥40%	Composite of total worsening HF events and death from cardiovascular causes	1083 primary-outcome events occurred in 624 of 3003 patients in the finerenone group vs 1283 in 719 of 2998 patients in the placebo group	RR: .84 (95% CI .74–.95); *P* = .007

If not otherwise specified, events and effect estimates refer to the primary endpoint.

### Sodium–glucose co-transporter-2 inhibitors

Sodium–glucose co-transporter 2 inhibitors (SGLT2i) were the next revolutionary step in the treatment of HF.^71,72^ First developed as glucose-lowering agents, SGLT2 inhibitors block glucose and sodium reabsorption in proximal convoluted tubules, leading to a transient diuretic effect.^18,73–75^ The first evidence for the cardioprotective effects of SGLT2 inhibitors came in 2015 from the EMPA-REG OUTCOME trial in patients with type 2 diabetes, where, compared with placebo, empagliflozin resulted in a reduction in the primary composite cardiovascular outcome, as well as in cardiovascular death and, interestingly, in hospitalizations for HF.^76^ This led to trials focusing on HF patients, regardless of diabetes status. First came RCTs in HF with reduced ejection fraction (HFrEF): DAPA-HF and EMPEROR-Reduced, comparing placebo with SGLT2 inhibitors on top of optimal medical therapy: dapagliflozin and empagliflozin, respectively.^77,78^ Both trials included patients with NYHA II-IV class HF and EF of 40% or less, with or without diabetes, and both showed a similar, ∼25% reduction in their primary composite endpoints (cardiovascular death and either HF worsening in DAPA-HF or hospitalization for HF in EMPEROR-Reduced, see *Table 4* and *Figure 5*) in patients on dapagliflozin and empagliflozin, respectively. In both trials, this effect was consistent regardless of diabetes status. Although the design of the two trials was somewhat different, focusing either on first HF worsening (DAPA-HF) or total HF hospitalizations (EMPEROR-Reduced), in both trials, SGLT2 inhibitors led to an about 30% reduction in those HF events, as well as to a slower decline in estimated glomerular filtration rate (eGFR).^77,78^ Additionally, in DAPA-HF, a significant 18% reduction in cardiovascular mortality was observed with dapagliflozin compared with placebo.^77^ However, a subsequent meta-analysis of the DAPA-HF and EMPEROR-reduced trials found no heterogeneity between the two agents and demonstrated a 14% reduction in cardiovascular mortality with SGLT2 inhibitors compared with placebo.^79^

**Table 4:** Clinical trials supporting the use of SGLT2 inhibitors in heart failure

Trial (year of publication)	Intervention	Number of patients	Special information about the population	Primary outcome	Events: intervention vs comparator	Effect measure
HFrEF						
DAPA-HF (2019)	Dapagliflozin vs placebo	4744	LVEF≤40% NYHA class II, III or IV with or without diabetes	CV death or worsening HF (HHF or an urgent visit for HF)	16% vs 21%	HR: .74 (95% CI .65–.85); *P* < .001
EMPEROR-Reduced (2020)	Empagliflozin vs placebo	3730	LVEF≤40% NYHA class II, III or IV with or without diabetes	CV death or HHF	19% vs 25%	HR: .75 (95% CI .65–.86); *P* < .001
HFpEF						
EMPEROR-Preserved (2021)	Empagliflozin vs placebo	5988	LVEF>40% NYHA class II, III or IV with or without diabetes	CV death or HHF	14% vs 17%	HR: .79 (95% CI .69–.90); *P* < .001
DELIVER (2022)	Dapagliflozin vs placebo	6263	LVEF>40% NYHA class II, III or IV with or without diabetes	CV death or worsening HF (HHF or an urgent visit for HF)	16% vs 20%	HR: .82 (95% CI .73–.92); *P* < .001

If not otherwise specified, events and effect estimates refer to the primary endpoint.

RCTs in HF with mildly reduced (HFmrEF) and preserved EF (HFpEF) followed, and included EMPEROR-preserved (empagliflozin) and DELIVER (dapagliflozin).^80,81^ Both enrolled patients with NYHA II-IV class HF and EF of more than 40%, with or without diabetes. Again, the two trials differed in their definition of HF events (hospitalization for HF in EMPEROR-Preserved and HF worsening in DELIVER). Still, they both resulted in a similar reduction in their primary composite endpoints (cardiovascular death and HF hospitalization/worsening) with SGTL2 inhibitors compared with placebo. The effect on the primary endpoints was consistent across diabetes status and was mainly driven by a lower risk of HF events (HF hospitalizations or HF worsening, depending on the trial) in patients receiving SGLT2 inhibitors compared with placebo. However, neither trial demonstrated a significant reduction in cardiovascular mortality.^80,81^ A subsequent meta-analysis of the two trials confirmed a 20% reduction in the composite of cardiovascular death and HF hospitalization, and a 26% reduction in HF hospitalization, with a trend for reduction in cardiovascular mortality (hazard ratio [HR] .88, 95% confidence interval .77–1.00; *P* = .052).^82^

Those landmark trials prompted a broadened recommendation for dapagliflozin and empagliflozin in HF across the whole EF spectrum.^44^ Thus, SGLT2 inhibitors (dapagliflozin or empagliflozin) became the first class to be indicated with class I recommendation in all patients with HF, irrespective of EF—including those with HFpEF and HFmrEF. Other trials, such as SOLOIST-WHF (with sotagliflozin, including only patients with type 2 diabetes) and EMPULSE (with empagliflozin), demonstrated benefits of initiating SGLT2 inhibitors early during/after HF decompensation.^83–85^

### Intravenous iron therapy

Following unsuccessful attempts to improve outcomes through correction of anaemia in HF, subsequent research shifted its focus towards iron deficiency (ID), which is one of the most common non-cardiac comorbidities in these patients.^86–89^ In a few large RCTs, beginning with the 2009 FAIR-HF trial, intravenous iron therapy was shown to improve symptoms, functional status, exercise capacity, and quality of life in patients with HFrEF and concomitant ID.^90^ However, evidence regarding hard clinical endpoints has been less consistent.^91^ Two large RCTs suggested a potential benefit, particularly regarding recurrent HF hospitalizations (*Table 5* and *Figure 5*). In the AFFIRM-AHF trial, among patients with HFrEF who were stabilized during the index acute HF episode, intravenous ferric carboxymaltose administered before discharge reduced total HF hospitalizations and CV death compared with placebo (RR .75; 95% CI .59–.96; *P* = .024) during the follow-up period in the COVID-19 sensitivity analysis.^92^ In the IRONMAN trial, intravenous ferric derisomaltose was associated with a lower risk of recurrent HF hospitalizations and cardiovascular death (RR .76; 95% CI .58–1.00; *P* = .047) (COVID-19-sensitive analysis).^93^ In contrast, two other large RCTs yielded neutral results.^94,95^ ESC guidelines (focused update from 2023) state that intravenous iron supplementation with ferric carboxymaltose or ferric derisomaltose should be considered in symptomatic patients with HFrEF or HFmrEF and ID to reduce the risk of HF hospitalization.^44^ More recent meta-analyses, including data from the latest RCTs, suggest that intravenous iron therapy may reduce recurrent HF hospitalizations and the composite of recurrent HF hospitalizations and cardiovascular mortality in patients with HFrEF and ID.^96^ There are no data regarding HFpEF from sufficiently powered RCTs.^97^

**Table 5:** Clinical trials supporting the use of intravenous iron, vericiguat, ivabradine, digitalis glycosides, and incretin-based therapies in heart failure

Trial (year of publication)	Intervention	Number of patients	Special information about the population	Primary outcome	Events: intervention vs comparator	Effect measure
Intravenous iron						
AFFIRM-AHF (2020)	Ferric carboxymaltose vs placebo	1525	Patients stabilized after an episode of AHF, iron deficient, LVEF < 50%	Total HF hospitalizations and CV death	COVID-19 sensitivity analysis: 274 vs 363	RR: .75 (95% CI .59–.96); *P* = .02
IRONMAN (2022)	Ferric derisomaltose vs placebo	1869	Iron deficient, LVEF≤45%	Recurrent hospital admissions for HF and CV death	COVID-19 sensitivity analysis: 210 vs 280	RR: .76 (95% CI .58–1.00); *P* = .047
Vericiguat						
VICTORIA (2020)	Vericiguat vs placebo	5050	HF with worsening in 6-3 months prior to randomization	Composite of CV death or first HF hospitalization	897 vs 932	HR: .90 (95% CI .82–.98); *P* = .02
VICTOR (2025)	Vericiguat vs placebo	6105	HF without worsening in 6-3 months prior to randomization	Composite of CV death or first HF hospitalization	549 vs 584	HR: .93 (95% CI .83–1.04); *P* = .22
Ivabradine						
SHIFT (2010)	Ivabradine vs placebo	6558	Symptomatic HFrEF (LVEF ≤ 35%) and SR ≥ 70/min and HHF within previous 12 months, on β-blocker if tolerated	Composite of CV death and hospital admission for worsening HF	793 (24%) vs 937 (29%)	HR: .82 (95% CI .75–.90); *P* < .001
Digitalis glycosides						
DIG (1997)	Digoxin vs placebo	6800	Sinus rhythm, LVEF≤45%	All-cause mortality	1181 (35%) vs 1194 (35%)	Risk ratio: .99 (95% CI .91–1.07); *P* = .80
DIGIT-HF (2025)	Digitoxin vs placebo	1212	LVEF≤40% & NYHA III-IV or LVEF≤30% & NYHA II; AFib 27%	Composite of death from any cause or hospital admission for worsening HF	242 (40%) vs 264 (44%)	HR: .82 (95% CI .69–.98); *P* = .03
Incretin-based therapies						
FIGHT (2016)	Liraglutide vs placebo	300	HFrEF (EF ≤ 40%) and recent HHF	Global rank score across 3 hierarchical tiers: time to death, time to rehospitalization for HF, and time-averaged proportional change in NT-proBNP	Mean rank of 146 vs 156	Wilcoxon rank sum *P* =.31
EXSCEL (2024)	Exenatide vs placebo	4749	T2D (*post-hoc* analysis of HF patients)	Time to first event of HHF	LVEF <40%: 20% vs 13%; LVEF ≥40%: 3% vs 5%	HR: 1.52 (95% CI .95–2.43); HR: .74 (95% CI .55–1.01)
SUMMIT (2024)	Tirzepatide vs placebo	731	Obesity (BMI >30 kg/m^2^) and HFpEF (EF ≥ 50%)	Composite of CV death or worsening HF	10% vs 15%	HR: .62 (95% CI .4–.95); *P* = .026

If not otherwise specified, events and effect estimates refer to the primary endpoint.

### Vericiguat

Vericiguat is a soluble guanylate cyclase stimulator and stabilizer located in the cytoplasm of vascular smooth muscle cells and cardiomyocytes; its activity is independent of NO (whose bioavailability in the failing myocardium is diminished). Increasing intracellular cGMP concentrations may improve myocardial energetics and contractile performance.

The first phase III trial, VICTORIA, was initiated in 2016 and published in 2020. A total of 5050 patients with HFrEF (LVEF <45%) and NYHA class II–IV symptoms were enrolled following recent WHF (hospitalization for HF within 6 months prior to randomization or outpatient administration of intravenous diuretics for HF within 3 months prior to randomization).^98^ The mean LVEF of the population was 29%; NYHA class II patients accounted for 59%–60%; and the median follow-up was 10.8 months. The patients were randomized to receive vericiguat or a placebo in addition to GDMT. A reduction in the primary composite endpoint (cardiovascular death or first hospitalization for HF) was demonstrated (*Table 5* and *Figure 5*). Death from cardiovascular causes occurred in 16.4% of patients in the vericiguat group and 17.5% in the placebo group (HR .93; 95% CI .81–1.06). Notably, the drug appeared to confer greater benefit in patients with baseline NT-proBNP levels not exceeding ∼6000 pg/ml, LVEF (<40%) and eGFR >30 ml/min/1.73 m^2^.

Based on this, another phase III RCT, the VICTOR trial, was conducted.^99^ It included 6105 ambulatory patients with chronic HFrEF. The trial applied similar inclusion criteria to VICTORIA but recruited stable, ambulatory HF patients without a requirement for recent worsening of HF. The mean LVEF was 30% and 80% were NYHA class II. No reduction in the primary endpoint was demonstrated. Cardiovascular and all-cause mortality were numerically lower in the vericiguat group; however, because statistical significance for the primary endpoint was not achieved, these findings should be considered exploratory rather than confirmatory. In a pooled analysis of these two studies, the primary endpoint of cardiovascular death or hospitalization for HF was reduced in the vericiguat group compared with placebo (HR .91 [95% CI .85–.98]; *P* = .0088), with similar reductions in its individual components of cardiovascular death (.89 [.80–.98]; *P* = .020) and hospitalization for HF (.92 [.84–1.00]; *P* = .043) as first events.^100^ No phase III RCTs have been conducted in HFpEF. SOCRATES-PRESERVED was a phase II RCT in patients with LVEF>45%. It did not demonstrate a reduction in NT-proBNP but was well tolerated.

### Ivabradine

The concept that heart rate reduction benefits the myocardium led to the introduction of ivabradine into the therapy of HFrEF. Ivabradine is an inhibitor of the I_f_ current in the sinoatrial node.^101^ Unlike β-blockers, ivabradine does not modify myocardial contractility and intracardiac conduction, even in patients with impaired systolic function.^102^ The SHIFT study was intended to evaluate the effect of HR reduction in HFrEF (LVEF ≤ 35%). Among 6558 HFrEF patients (NYHA II-IV), ischemic aetiology was present in 4418 (68%). Ivabradine or placebo were used on top of standard therapy with RAAS antagonist (91% patients) and β blocker (89%). Treatment with ivabradine was associated with 18% lower risk of the primary endpoint.^103^ Ivabradine was also associated with an average reduction in heart rate of 15 b.p.m. from a baseline value of 80 b.p.m. Based on these results, ivabradine received class IIa recommendation for the treatment of symptomatic HFrEF patients with sinus rhythm ≥ 70 b.p.m., on maximum tolerated β blocker (or without β blocker if not tolerated or contraindicated), ACE-I/ARNI and MRA.^5^ However, the European Medicines Agency approved ivabradine for use in Europe in patients with HFrEF with LVEF ≤ 35% and in SR ≥75 b.p.m., because in this group ivabradine was associated with survival benefit in a secondary subgroup analysis of the SHIFT study^104^ (*Table 5* and *Figure 5*). In one small trial regarding symptomatic HFpEF patients with heart rate of 70 b.p.m. or more, HR reduction with ivabradine did not translate into better outcomes.^105^

### Digitalis glycosides

Digitalis glycosides are among the earliest pharmacological agents systematically used in the treatment of cardiovascular disease. In HF, the evidence base for their clinical use is derived primarily from the Digitalis Investigation Group trial.^106^ In this pivotal randomized trial, 6800 patients with LVEF≤45% were assigned to digoxin or placebo in addition to diuretics and ACEI and followed for a mean of over 3 years. Although digoxin did not reduce all-cause mortality, it significantly decreased hospitalizations due to HF as well as overall hospitalizations.^106^ In an ancillary study including patients with HF with preserved ejection fraction LVEF>45%, digoxin had no significant effect on major clinical endpoints compared with placebo.^107^ More recently, in the DIGIT-HF trial, which enrolled 1212 contemporary patients with symptomatic HFrEF, digitoxin reduced the composite risk of all-cause mortality or hospitalization for worsening HF compared with placebo.^108^

### Incretin-based therapies

The first data on the effect of glucagon-like peptide-1 receptor agonists (GLP-1 RAs) on cardiovascular mortality came from studies in patients with type 2 diabetes mellitus (T2DM) which revealed that GLP-1 RAs decreased the relative risk of major adverse cardiovascular events by 14%.^109^

The effects of GLP-1 RAs in HF are less clear, and studies designed specifically to evaluate patients with HF are scarce (*Table 5* and *Figure 5*). The meta-analysis, which included 54 092 patients with T2DM, showed that treatment with GLP-1 RA did not reduce HF hospitalizations and mortality in patients with concomitant T2DM and HF, but may be preventive of new-onset HF and mortality in patients with T2DM without HF. The reduction of atherosclerotic events with GLP-1 RA was not influenced by HF history status.^110^ These results should be interpreted in the context that patients with advanced HF, recent decompensation, or severely reduced LVEF were excluded from these trials.

Five RCTs were designed specifically to evaluate HF population, with the STEP-HFpEF, STEP-HFpEF DM and SUMMIT trials focusing on HFpEF (*Table 5*). The STEP-HFpEF and STEP-HFpEF DM trials showed significantly higher functional capacity and greater weight loss in obese HFpEF patients treated with semaglutide vs placebo irrespective of concomitant T2DM.^111,112^ A pre-specified pooled analysis of individual patient data from these studies (*n* = 1145), although still underpowered, evaluated a composite of death from any cause, number of HF events, and timing of HF.^113^ A significantly greater number of wins for the hierarchical composite endpoint (win ratio 1.65 [1.42–1.91]; *P* < .0001) was observed for semaglutide vs placebo, which provides a rationale for further assessment of semaglutide in dedicated outcome trials.

Tirzepatide is a long-acting agonist of glucose-dependent insulinotropic polypeptide and GLP-1 receptors that results in 12% to 21% weight loss in patients with obesity.^114,115^ Treatment with tirzepatide led to a 38% lower risk of the primary composite of CV death or worsening HF event vs placebo and improved health status in patients with HFpEF and obesity (SUMMIT trial).^116^

Two RCTs targeted specifically HFrEF patients: the FIGHT and the LIVE trial. The FIGHT trial was designed to evaluate liraglutide in a high-risk population of HFrEF defined as EF ≤ 40%, within 14 days of hospitalization for an acute HF despite evidence-based therapy. Compared with placebo, liraglutide had no significant effect on the primary endpoint (*Table 5*).^117^ In a *post-hoc* analysis of the FIGHT trial, there was an increased risk of hospitalization for HF (HHF) (124 vs 80 events; *P* = .061) and arrhythmic events (39 vs 21 events; *P* = .088), particularly among patients with a NYHA class of III/IV.^118^

In the LIVE trial, which focused on a different population of clinically stable HFrEF patients receiving optimal medical treatment, there was an increased risk of serious cardiac events with liraglutide compared with placebo (12 [10%] vs 3 [3%] events, *P* = .04), driven by ventricular arrhythmia (four events, including 1 death, vs 1 event) and atrial fibrillation requiring intervention (four vs two events).^119^

In the EXSCEL trial on exenatide in T2DM data on LVEF were collected, providing the opportunity to evaluate the effect of GLP-1 RA on cardiovascular outcomes according to LVEF. The exenatide effect on HF hospitalization was influenced by LVEF, with a potentially decreased risk in participants with LVEF ≥40% and increased risk in those with LVEF <40%. The risk of HHF was particularly high in participants with LVEF <40% and NYHA class III/IV. LVEF did not modify the effect of exenatide on atherosclerotic outcomes.^120^ The results are consistent with the SELECT trial including obese patients without T2DM which showed a similar effect of LVEF on HFH.^121^

In summary, the most robust evidence for incretin-based therapies in HF currently pertains to the phenotype of obesity and HFpEF.

### Diversity of care and implementation schemes

Despite established core guidelines shaping foundational pharmacotherapy in HF, several therapeutic questions remain, and answers to them are gradually emerging. Among the issues requiring particular attention is the inequality in access to GDMT, and registry-based studies should provide further insights into this problem.^122–125^ The strategy of rapid, simultaneous initiation of therapy has been evaluated in large clinical trials and has proven beneficial with respect to the pre-defined endpoints.^19,41,71,126–128^ It is also important to emphasize the representativeness of specific patient groups in clinical studies, including women, the elderly, socioeconomically disadvantaged individuals, and patients with advanced comorbidities.^125,129–133^ The maturity of healthcare systems will be reflected in their ability to adequately include these vulnerable populations. Furthermore, optimal pharmacotherapy in cases of drug intolerance or in patients treated with modern left ventricular assist device armamentarium—such as medium-term microaxial pumps (cardiogenic shock due to MI, severe HF pre-transplant, acute fulminant myocarditis)—remains insufficiently defined and is not yet supported by clear guideline recommendations.^134,135^

## Conclusions

Contemporary HF pharmacotherapy reflects decades of clinical investigation, with landmark RCTs establishing robust evidence base for disease-modifying therapies that alter the natural history of the condition. Particularly in HFrEF, the integration of multiple drug classes targeting complementary pathophysiological mechanisms—including neurohormonal activation, sympathetic overactivity, and the disordered cardiorenal axis—has translated into substantial improvements in survival and morbidity. Despite this strong evidence base, a persistent gap remains between guideline recommendations and real-world implementation. Bridging this gap should be a key priority, alongside improving representation of under-represented populations in clinical trials. Future research should also focus on the development of novel therapeutic strategies and the advancement of precision medicine approaches to enable more individualized treatment of patients with HF.

## Declarations

### Data Availability

This article did not involve the generation or analysis of any original data. Therefore, no data are available for sharing.

## References

[1] Vogl A . The discovery of the organic mercurial diuretics. Am Heart J 1950;39:881–3. 10.1016/0002-8703(50)90299-7 15419149

[2] Reubi FC . Clinical use of furosemide. Ann N Y Acad Sci 1966;139:433–42. 10.1111/j.1749-6632.1966.tb41217.x 5230286

[3] Huang X, Dorhout Mees E, Vos P, Hamza S, Braam B. Everything we always wanted to know about furosemide but were afraid to ask. Am J Physiol Renal Physiol 2016;310:F958–71. 10.1152/ajprenal.00476.2015 26911852

[4] Mullens W, Damman Kevin, Harjola V-P, Mebazaa A, Brunner-La Rocca H-P, Martens P, et al The use of diuretics in heart failure with congestion - a position statement from the Heart Failure Association of the European Society of Cardiology. Eur J Heart Fail 2019;21:137–55. 10.1002/ejhf.1369 30600580

[5] McDonagh TA, Metra M, Adamo M, Gardner RS, Baumbach A, Böhm M, et al 2021 ESC guidelines for the diagnosis and treatment of acute and chronic heart failure. Eur Heart J 2021;42:3599–726. 10.1093/eurheartj/ehab368 34922348

[6] Heidenreich PA, Bozkurt B, Aguilar D, Allen LA, Byun JJ, Colvin MM, et al 2022 AHA/ACC/HFSA guideline for the management of heart failure: a report of the American College of Cardiology/American Heart Association Joint Committee on clinical practice guidelines. Circulation 2022;145:e895–1032. 10.1161/CIR.0000000000001063 35363499

[7] Pinto FJ, Anker SD, Abraham WT, Atherton JJ, Butler J, Chopra V, et al The global implementation guidelines initiative: how to optimize cardio-renal-metabolic care worldwide. Glob Cardiol 2025;3:37–41. 10.4081/cardio.2025.68

[8] Chopra V, Zieroth S. iCARDIO alliance global implementation guidelines on heart failure 2025. Glob Cardiol 2025;3:47–74. 10.4081/cardio.2025.70 40778878

[9] Naidu AS, Ambrosy AP, Cotter G, Bocchi EA, Butler J, Chioncel O, et al Early in-hospital treatment of acute heart failure. Part 2 of the international expert opinion series on AHF management. ESC Heart Fail 2025;12:3826–43. 10.1002/ehf2.15389 41014641 PMC12719859

[10] Palazzuoli A, Del Buono MG, La Vecchia G, Greene SJ, Ambrosy AP, Chioncel O, et al Worsening versus advanced heart failure: management and challenges. ESC Heart Fail 2025;12:3856–68. 10.1002/ehf2.15437 41045229 PMC12719834

[11] Cobo Marcos M, de la Espriella R, Zegri-Reiriz I, Llacer P, Rubio Gracia J, Comín-Colet J, et al Early diuretic response and outcome prediction in ambulatory worsening heart failure: natriuresis versus diuresis. ESC Heart Fail 2025;12:2460–6. 10.1002/ehf2.15275 40152188 PMC12287789

[12] Biegus J, Zymlinski R, Ponikowski P. Loop diuretics in heart failure: the objective markers to guide the therapy are needed. ESC Heart Fail 2024;11:1816–8. 10.1002/ehf2.14920 38923785 PMC11287360

[13] Croset F, Llàcer P, Núñez J, Campos J, García M, Pérez A, et al Loop diuretic down-titration at discharge in patients hospitalized for acute heart failure. ESC Heart Fail 2024;11:1739–47. 10.1002/ehf2.14749 38454739 PMC11098660

[14] Shiraishi Y, Kurita Y, Mori H, Ooishi K, Matsukawa M. Time to intravenous diuretic administration in patients hospitalized with heart failure: an observational study. ESC Heart Fail 2024;11:4061–70. 10.1002/ehf2.15005 39105376 PMC11631256

[15] Baudry G, Pereira O, Duarte K, Ferreira JP, Savarese G, Welter A, et al Risk of readmission and death after hospitalization for worsening heart failure: Role of post-discharge follow-up visits in a real-world study from the Grand Est Region of France. Eur J Heart Fail 2024;26:342–54. 10.1002/ejhf.3103 38059342

[16] Baudry G, Pereira O, Roubille F, Villaceque M, Damy T, Duarte K, et al Cardiologist follow-up and improved outcomes of heart failure: a French nationwide cohort. Eur Heart J 2025;46:3050–65. 10.1093/eurheartj/ehaf218 40382685 PMC12349951

[17] Friday JM, Cleland JGF, Pellicori P, Wolters MK, McMurray JJV, Jhund PS, et al Loop diuretic therapy with or without heart failure: impact on prognosis. Eur Heart J 2024;45:3837–49. 10.1093/eurheartj/ehae345 38845446 PMC11452746

[18] Biegus J, Cotter G, Metra M, Ponikowski P. Decongestion in acute heart failure: is it time to change diuretic-centred paradigm? Eur J Heart Fail 2024;26:2094–106. 10.1002/ejhf.3423 39169731

[19] Biegus J, Mebazaa A, Davison B, Cotter G, Edwards C, Čelutkienė J, et al Effects of rapid uptitration of neurohormonal blockade on effective, sustainable decongestion and outcomes in STRONG-HF. J Am Coll Cardiol 2024;84:323–36. 10.1016/j.jacc.2024.04.055 39019527

[20] Biegus J, Cotter G, Davison BA, Metra M, Pagnesi M, Ponikowski P. Diuretic de-escalation in response to HF therapy: are we closer to reduced reliance on diuretics? JACC Heart Fail 2025;13:846–54. 10.1016/j.jchf.2025.03.010 40335234

[21] CONSENSUS Trial Study Group . Effects of enalapril on mortality in severe congestive heart failure. Results of the Cooperative North Scandinavian Enalapril Survival Study (CONSENSUS). N Engl J Med 1987;316:1429–35. 10.1056/NEJM198706043162301 2883575

[22] Yusuf S, Pitt B, Davis CE, Hood WB, Cohn JN. Effect of enalapril on survival in patients with reduced left ventricular ejection fractions and congestive heart failure. N Engl J Med 1991;325:293–302. 10.1056/NEJM199108013250501 2057034

[23] Pfeffer MA, Braunwald E, Moyé LA, Basta L, Brown EJ, Cuddy TE, et al Effect of captopril on mortality and morbidity in patients with left ventricular dysfunction after myocardial infarction. Results of the survival and ventricular enlargement trial. The SAVE Investigators. N Engl J Med 1992;327:669–77. 10.1056/NEJM199209033271001 1386652

[24] Effect of ramipril on mortality and morbidity of survivors of acute myocardial infarction with clinical evidence of heart failure. The Acute Infarction Ramipril Efficacy (AIRE) Study Investigators. Lancet 1993;342:821–8. 8104270

[25] Køber L, Torp-Pedersen C, Carlsen JE, Bagger H, Eliasen P, Lyngborg K, et al A clinical trial of the angiotensin-converting-enzyme inhibitor trandolapril in patients with left ventricular dysfunction after myocardial infarction. Trandolapril Cardiac Evaluation (TRACE) Study Group. N Engl J Med 1995;333:1670–6. 10.1056/NEJM199512213332503 7477219

[26] Packer M, Poole-Wilson PA, Armstrong PW, Cleland JG, Horowitz JD, Massie BM, et al Comparative effects of low and high doses of the angiotensin-converting enzyme inhibitor, lisinopril, on morbidity and mortality in chronic heart failure. ATLAS Study Group. Circulation 1999;100:2312–8. 10.1161/01.CIR.100.23.2312 10587334

[27] Cleland JG, Tendera M, Adamus J, Freemantle N, Polonski L, Taylor J. The perindopril in elderly people with chronic heart failure (PEP-CHF) study. Eur Heart J 2006;27:2338–45. 10.1093/eurheartj/ehl250 16963472

[28] Pitt B, Poole-Wilson PA, Segal R, Martinez FA, Dickstein K, Camm AJ, et al Effect of losartan compared with captopril on mortality in patients with symptomatic heart failure: randomised trial–the Losartan Heart Failure Survival Study ELITE II. Lancet 2000;355:1582–7. 10.1016/S0140-6736(00)02213-3 10821361

[29] Cohn JN, Tognoni G. A randomized trial of the angiotensin-receptor blocker valsartan in chronic heart failure. N Engl J Med 2001;345:1667–75. 10.1056/NEJMoa010713 11759645

[30] Granger CB, McMurray JJV, Yusuf S, Held P, Michelson EL, Olofsson B, et al Effects of candesartan in patients with chronic heart failure and reduced left-ventricular systolic function intolerant to angiotensin-converting-enzyme inhibitors: the CHARM-alternative trial. Lancet 2003;362:772–6. 10.1016/S0140-6736(03)14284-5 13678870

[31] McMurray JJ, Ostergren Jan, Swedberg K, Granger CB, Held P, Michelson EL, et al Effects of candesartan in patients with chronic heart failure and reduced left-ventricular systolic function taking angiotensin-converting-enzyme inhibitors: the CHARM-added trial. Lancet 2003;362:767–71. 10.1016/S0140-6736(03)14283-3 13678869

[32] Pfeffer MA, McMurray JJV, Velazquez EJ, Rouleau J-L, Køber L, Maggioni AP, et al Valsartan, captopril, or both in myocardial infarction complicated by heart failure, left ventricular dysfunction, or both. N Engl J Med 2003;349:1893–906. 10.1056/NEJMoa032292 14610160

[33] Dickstein K, Kjekshus J. Effects of losartan and captopril on mortality and morbidity in high-risk patients after acute myocardial infarction: the OPTIMAAL randomised trial. Optimal trial in myocardial infarction with angiotensin ii antagonist losartan. Lancet 2002;360:752–60. 10.1016/S0140-6736(02)09895-1 12241832

[34] Konstam MA, Neaton JD, Dickstein K, Drexler H, Komajda M, Martinez FA, et al Effects of high-dose versus low-dose losartan on clinical outcomes in patients with heart failure (HEAAL study): a randomised, double-blind trial. Lancet 2009;374:1840–8. 10.1016/S0140-6736(09)61913-9 19922995

[35] Yusuf S, Pfeffer MA, Swedberg K, Granger CB, Held P, McMurray JJV, et al Effects of candesartan in patients with chronic heart failure and preserved left-ventricular ejection fraction: the CHARM-preserved trial. Lancet 2003;362:777–81. 10.1016/S0140-6736(03)14285-7 13678871

[36] Massie BM, Carson PE, McMurray JJ, Komajda M, McKelvie R, Zile MR, et al Irbesartan in patients with heart failure and preserved ejection fraction. N Engl J Med 2008;359:2456–67. 10.1056/NEJMoa0805450 19001508

[37] McMurray JJ, Packer M, Desai AS, Gong J, Lefkowitz MP, Rizkala AR, et al Angiotensin-neprilysin inhibition versus enalapril in heart failure. N Engl J Med 2014;371:993–1004. 10.1056/NEJMoa1409077 25176015

[38] Velazquez EJ, Morrow DA, DeVore AD, Duffy CI, Ambrosy AP, McCague K, et al Angiotensin-neprilysin inhibition in acute decompensated heart failure. N Engl J Med 2019;380:539–48. 10.1056/NEJMoa1812851 30415601

[39] Wachter R, Senni M, Belohlavek J, Straburzynska-Migaj E, Witte KK, Kobalava Z, et al Initiation of sacubitril/valsartan in haemodynamically stabilised heart failure patients in hospital or early after discharge: primary results of the randomised TRANSITION study. Eur J Heart Fail 2019;21:998–1007. 10.1002/ejhf.1498 31134724

[40] Rajzer P, Biegus J. Sacubitril/valsartan in a wide spectrum of heart failure patients (from mechanisms of action to outcomes in specific populations). Heart Fail Rev 2025;30:387–405. 10.1007/s10741-024-10471-1 39776087 PMC11802626

[41] Ferreira DR, Cazeiro DI, Brito J, Santos R, Rigueira J, Silva D, et al Impact of early intensive GDMT on LVEF recovery and ICD decision making in de Novo HFrEF. ESC Heart Fail 2025;12:4299–304. 10.1002/ehf2.15443 41206800 PMC12719864

[42] Solomon SD, McMurray JJV, Anand IS, Ge J, Lam CSP, Maggioni AP, et al Angiotensin-neprilysin inhibition in heart failure with preserved ejection fraction. N Engl J Med 2019;381:1609–20. 10.1056/NEJMoa1908655 31475794

[43] Solomon SD, Vaduganathan M, L Claggett B, Packer M, Zile M, Swedberg K, et al Sacubitril/valsartan across the Spectrum of ejection fraction in heart failure. Circulation 2020;141:352–61. 10.1161/CIRCULATIONAHA.119.044586 31736342

[44] McDonagh TA, Metra M, Adamo M, Gardner RS, Baumbach A, Böhm M, et al 2023 focused update of the 2021 ESC guidelines for the diagnosis and treatment of acute and chronic heart failure. Eur Heart J 2023;44:3627–39. 10.1093/eurheartj/ehad195 37622666

[45] Effect of metoprolol CR/XL in chronic heart failure: metoprolol CR/XL randomised intervention trial in congestive heart failure (MERIT-HF). Lancet 1999;353:2001–7. 10.1016/S0140-6736(99)04440-2 10376614

[46] The cardiac insufficiency bisoprolol study II (CIBIS-II): a randomised trial. Lancet 1999;353:9–13. 10.1016/S0140-6736(98)11181-9 10023943

[47] Packer M, Coats AJ, Fowler MB, Katus HA, Krum H, Mohacsi P, et al Effect of carvedilol on survival in severe chronic heart failure. N Engl J Med 2001;344:1651–8. 10.1056/NEJM200105313442201 11386263

[48] Flather MD, Shibata MC, Coats AJS, Van Veldhuisen DJ, Parkhomenko A, Borbola J, et al Randomized trial to determine the effect of nebivolol on mortality and cardiovascular hospital admission in elderly patients with heart failure (SENIORS). Eur Heart J 2005;26:215–25. 10.1093/eurheartj/ehi115 15642700

[49] Dargie HJ . Effect of carvedilol on outcome after myocardial infarction in patients with left-ventricular dysfunction: the CAPRICORN randomised trial. Lancet 2001;357:1385–90. 10.1016/S0140-6736(00)04560-8 11356434

[50] Packer M, Bristow MR, Cohn JN, Colucci WS, Fowler MB, Gilbert EM, et al The effect of carvedilol on morbidity and mortality in patients with chronic heart failure. U.S. Carvedilol Heart Failure Study Group. N Engl J Med 1996;334:1349–55. 10.1056/NEJM199605233342101 8614419

[51] Eichhorn EJ, Domanski MJ, Krause-Steinrauf H, Bristow MR, Lavori PW. A trial of the beta-blocker bucindolol in patients with advanced chronic heart failure. N Engl J Med 2001;344:1659–67. 10.1056/NEJM200105313442202 11386264

[52] Foody JM, Farrell MH, Krumholz HM. beta-blocker therapy in heart failure: scientific review. JAMA 2002;287:883–9. 10.1001/jama.287.7.883 11851582

[53] Cleophas TJ, Zwinderman AH. Beta-blockers and heart failure: meta-analysis of mortality trials. Int J Clin Pharmacol Ther 2001;39:383–8. 10.5414/CPP39383 11563684

[54] Waagstein F, Bristow MR, Swedberg K, Camerini F, Fowler MB, Silver MA, et al Beneficial effects of metoprolol in idiopathic dilated cardiomyopathy. Metoprolol in Dilated Cardiomyopathy (MDC) Trial Study Group. Lancet 1993;342:1441–6. 10.1016/0140-6736(93)92930-R 7902479

[55] A randomized trial of beta-blockade in heart failure. The Cardiac Insufficiency Bisoprolol Study (CIBIS). CIBIS Investigators and Committees. Circulation 1994;90:1765–73. 10.1161/01.CIR.90.4.1765 7923660

[56] Al-Gobari M, El Khatib C, Pillon F, Gueyffier F. beta-blockers for the prevention of sudden cardiac death in heart failure patients: a meta-analysis of randomized controlled trials. BMC Cardiovasc Disord 2013;13:52. 10.1186/1471-2261-13-52 23848972 PMC3716800

[57] Cleland JGF, Bunting KV, Flather MD, Altman DG, Holmes J, Coats AJS, et al Beta-blockers for heart failure with reduced, mid-range, and preserved ejection fraction: an individual patient-level analysis of double-blind randomized trials. Eur Heart J 2018;39:26–35. 10.1093/eurheartj/ehx564 29040525 PMC5837435

[58] van Veldhuisen DJ, Cohen-Solal A, Böhm M, Anker SD, Babalis D, Roughton M, et al Beta-blockade with nebivolol in elderly heart failure patients with impaired and preserved left ventricular ejection fraction: data from SENIORS (study of effects of nebivolol intervention on outcomes and rehospitalization in seniors with heart failure). J Am Coll Cardiol 2009;53:2150–8. 10.1016/j.jacc.2009.02.046 19497441

[59] Kotecha D, Holmes J, Krum H, Altman DG, Manzano L, Cleland JGF, et al Efficacy of β blockers in patients with heart failure plus atrial fibrillation: an individual-patient data meta-analysis. Lancet 2014;384:2235–43. 10.1016/S0140-6736(14)61373-8 25193873

[60] Bavishi C, Chatterjee Saurav, Ather Sameer, Patel Dipen, Messerli Franz H. Beta-blockers in heart failure with preserved ejection fraction: a meta-analysis. Heart Fail Rev 2015;20:193–201. 10.1007/s10741-014-9453-8 25034701

[61] Yamamoto K, Origasa H, Hori M. Effects of carvedilol on heart failure with preserved ejection fraction: the Japanese Diastolic Heart Failure Study (J-DHF). Eur J Heart Fail 2013;15:110–8. 10.1093/eurjhf/hfs141 22983988

[62] Conraads VM, Metra M, Kamp O, De Keulenaer GW, Pieske B, Zamorano J, et al Effects of the long-term administration of nebivolol on the clinical symptoms, exercise capacity, and left ventricular function of patients with diastolic dysfunction: results of the ELANDD study. Eur J Heart Fail 2012;14:219–25. 10.1093/eurjhf/hfr161 22147202

[63] Palau P, Seller J, Domínguez E, Sastre C, Ramón JM, de La Espriella R, et al Effect of β-blocker withdrawal on functional capacity in heart failure and preserved ejection fraction. J Am Coll Cardiol 2021;78:2042–56. 10.1016/j.jacc.2021.08.073 34794685

[64] Pitt B, Zannad F, Remme WJ, Cody R, Castaigne A, Perez A, et al The effect of spironolactone on morbidity and mortality in patients with severe heart failure. N Engl J Med 1999;341:709–17. 10.1056/NEJM199909023411001 10471456

[65] Pitt B, Remme W, Zannad F, Neaton J, Martinez F, Roniker B, et al Eplerenone, a selective aldosterone blocker, in patients with left ventricular dysfunction after myocardial infarction. N Engl J Med 2003;348:1309–21. 10.1056/NEJMoa030207 12668699

[66] Zannad F, McMurray JJV, Krum H, van Veldhuisen DJ, Swedberg K, Shi H, et al Eplerenone in patients with systolic heart failure and mild symptoms. N Engl J Med 2011;364:11–21. 10.1056/NEJMoa1009492 21073363

[67] Pitt B, Pfeffer MA, Assmann SF, Boineau R, Anand IS, Claggett B, et al Spironolactone for heart failure with preserved ejection fraction. N Engl J Med 2014;370:1383–92. 10.1056/NEJMoa1313731 24716680

[68] Pfeffer MA, Claggett B, Assmann SF, Boineau R, Anand IS, Clausell N, et al Regional variation in patients and outcomes in the treatment of preserved cardiac function heart failure with an aldosterone antagonist (TOPCAT) Trial. Circulation 2015;131:34–42. 10.1161/CIRCULATIONAHA.114.013255 25406305

[69] Solomon SD, McMurray JJV, Vaduganathan M, Claggett B, Jhund PS, Desai AS, et al Finerenone in heart failure with mildly reduced or preserved ejection fraction. N Engl J Med 2024;391:1475–85. 10.1056/NEJMoa2407107 39225278

[70] Jhund PS, Talebi A, Henderson AD, Claggett BL, Vaduganathan M, Desai AS, et al Mineralocorticoid receptor antagonists in heart failure: an individual patient level meta-analysis. Lancet 2024;404:1119–31. 10.1016/S0140-6736(24)01733-1 39232490

[71] Beghini A, Sammartino AM, Papp Z, von Haehling S, Biegus J, Ponikowski P, et al 2024 update in heart failure. ESC Heart Fail 2025;12:8–42. 10.1002/ehf2.14857 38806171 PMC11769673

[72] Metra M, Tomasoni D, Adamo M, Amir O, Anker SD, Bayes-Genis A, et al SGLT2 inhibitors for the prevention and treatment of heart failure: a scientific statement of the HFA and the HFAI. ESC Heart Fail 2025;12:3806–25. 10.1002/ehf2.15408 40968746 PMC12719827

[73] Biegus J, Fudim M, Salah HM, Heerspink HJL, Voors AA, Ponikowski P. Sodium-glucose cotransporter-2 inhibitors in heart failure: potential decongestive mechanisms and current clinical studies. Eur J Heart Fail 2023;25:1526–36. 10.1002/ejhf.2967 37477086

[74] Biegus J, Voors AA, Collins SP, Kosiborod MN, Teerlink JR, Angermann CE, et al Impact of empagliflozin on decongestion in acute heart failure: the EMPULSE trial. Eur Heart J 2023;44:41–50. 10.1093/eurheartj/ehac530 36254693 PMC9805406

[75] Guzik M, Zymliński R, Biegus J. (February 18, 2026) Sodium-glucose cotransporter-2 inhibitors in heart failure. How durable is the decongestive effect? Eur J Heart Fail, 10.1093/ejhf/xuaf019 41771145

[76] Zinman B, Wanner C, Lachin JM, Fitchett D, Bluhmki E, Hantel S, et al Empagliflozin, cardiovascular outcomes, and mortality in type 2 diabetes. N Engl J Med 2015;373:2117–28. 10.1056/NEJMoa1504720 26378978

[77] McMurray JJV, Solomon SD, Inzucchi SE, Køber L, Kosiborod MN, Martinez FA, et al Dapagliflozin in patients with heart failure and reduced ejection fraction. N Engl J Med 2019;381:1995–2008. 10.1056/NEJMoa1911303 31535829

[78] Packer M, Anker SD, Butler J, Filippatos G, Pocock SJ, Carson P, et al Cardiovascular and renal outcomes with empagliflozin in heart failure. N Engl J Med 2020;383:1413–24. 10.1056/NEJMoa2022190 32865377

[79] Zannad F, Ferreira JP, Pocock SJ, Anker SD, Butler J, Filippatos G, et al SGLT2 inhibitors in patients with heart failure with reduced ejection fraction: a meta-analysis of the EMPEROR-reduced and DAPA-HF trials. Lancet 2020;396:819–29. 10.1016/S0140-6736(20)31824-9 32877652

[80] Anker SD, Butler J, Filippatos G, Ferreira JP, Bocchi E, Böhm M, et al Empagliflozin in heart failure with a preserved ejection fraction. N Engl J Med 2021;385:1451–61. 10.1056/NEJMoa2107038 34449189

[81] Solomon SD, McMurray JJV, Claggett B, de Boer RA, DeMets D, Hernandez AF, et al Dapagliflozin in heart failure with mildly reduced or preserved ejection fraction. N Engl J Med 2022;387:1089–98. 10.1056/NEJMoa2206286 36027570

[82] Vaduganathan M, Docherty KF, Claggett BL, Jhund PS, de Boer RA, Hernandez AF, et al SGLT-2 inhibitors in patients with heart failure: a comprehensive meta-analysis of five randomised controlled trials. Lancet 2022;400:757–67. 10.1016/S0140-6736(22)01429-5 36041474

[83] Voors AA, Angermann CE, Teerlink JR, Collins SP, Kosiborod M, Biegus J, et al The SGLT2 inhibitor empagliflozin in patients hospitalized for acute heart failure: a multinational randomized trial. Nat Med 2022;28:568–74. 10.1038/s41591-021-01659-1 35228754 PMC8938265

[84] Bhatt DL, Szarek M, Steg PG, Cannon CP, Leiter LA, McGuire DK, et al Sotagliflozin in patients with diabetes and recent worsening heart failure. N Engl J Med 2021;384:117–28. 10.1056/NEJMoa2030183 33200892

[85] Fioretti F, Butler J, Udell JA, Schuyler Jones W, Petrie MC, Harrington J, et al Empagliflozin after myocardial infarction with or without diabetes and chronic kidney disease: insights from EMPACT-MI. ESC Heart Fail 2025;12:3940–52. 10.1002/ehf2.15393 40947857 PMC12719805

[86] Lanser L, Pölzl G, Messner M, Ungericht M, Zaruba M-M, Hirsch J, et al Prevalence of iron deficiency in acute and chronic heart failure according to different clinical definitions. ESC Heart Fail 2025;12:1606–19. 10.1002/ehf2.15170 39930934 PMC12055403

[87] Sharma S, Katz R, Chaves PHM, Hoofnagle AN, Kizer JR, Bansal N, et al Iron deficiency and incident heart failure in older community-dwelling individuals. ESC Heart Fail 2024;11:1435–42. 10.1002/ehf2.14724 38407565 PMC11098627

[88] Aland SC, Gertler C, Bräunig HL, Schröder T, Edelmann F, Wachter R, et al Exercise capacity, iron deficiency and depressive symptoms in patients with asymptomatic chronic systolic heart failure. Glob Cardiol 2024;2:117–24. 10.4081/cardio.2024.39

[89] Akintunde AA, Orugun ST. The association of iron deficiency with right ventricular dysfunction in Africans with heart failure. Glob Cardiol 2024;2:184–90. 10.4081/cardio.2024.53

[90] von Haehling S . Iron deficiency in heart failure: epidemiology, diagnostic criteria and treatment modalities. ESC Heart Fail 2025;12:723–6. 10.1002/ehf2.15157 39474945 PMC11911631

[91] Ahmed M, Shafiq A, Javaid H, Singh P, Shahbaz H, Maniya MT, et al Intravenous iron therapy for heart failure and iron deficiency: an updated meta-analysis of randomized clinical trials. ESC Heart Fail 2025;12:43–53. 10.1002/ehf2.14905 38965691 PMC11769671

[92] Ponikowski P, Kirwan B-A, Anker SD, McDonagh T, Dorobantu M, Drozdz J, et al Ferric carboxymaltose for iron deficiency at discharge after acute heart failure: a multicentre, double-blind, randomised, controlled trial. Lancet 2020;396:1895–904. 10.1016/S0140-6736(20)32339-4 33197395

[93] Kalra PR, Cleland JGF, Petrie MC, Thomson EA, Kalra PA, Squire IB, et al Intravenous ferric derisomaltose in patients with heart failure and iron deficiency in the UK (IRONMAN): an investigator-initiated, prospective, randomised, open-label, blinded-endpoint trial. Lancet 2022;400:2199–209. 10.1016/S0140-6736(22)02083-9 36347265

[94] Karakas M, Friede T, Butler J, Talha KM, Placzek M, Asendorf T, et al Intravenous ferric carboxymaltose in heart failure with iron deficiency (FAIR-HF2 DZHK05 trial): sex-specific outcomes. Eur J Heart Fail 2025;27:2328–42. 10.1002/ejhf.3742 40740027 PMC12765045

[95] Mentz RJ, Garg J, Rockhold FW, Butler J, De Pasquale CG, Ezekowitz JA, et al Ferric carboxymaltose in heart failure with iron deficiency. N Engl J Med 2023;389:975–86. 10.1056/NEJMoa2304968 37632463

[96] Anker SD, Karakas M, Mentz RJ, Ponikowski P, Butler J, Khan MS, et al Systematic review and meta-analysis of intravenous iron therapy for patients with heart failure and iron deficiency. Nat Med 2025;31:2640–6. 10.1038/s41591-025-03671-1 40159279 PMC12353798

[97] Khan LA, Javaid SS, Butler J. Iron supplementation in heart failure and iron deficiency: does it help? Glob Cardiol 2025;3:159–63. 10.4081/cardio.2025.82

[98] Armstrong PW, Pieske B, Anstrom KJ, Ezekowitz J, Hernandez AF, Butler J, et al Vericiguat in patients with heart failure and reduced ejection fraction. N Engl J Med 2020;382:1883–93. 10.1056/NEJMoa1915928 32222134

[99] Butler J, McMullan CJ, Anstrom KJ, Barash I, Bonaca MP, Borentain M, et al Vericiguat in patients with chronic heart failure and reduced ejection fraction (VICTOR): a double-blind, placebo-controlled, randomised, phase 3 trial. Lancet 2025;406:1341–50. 10.1016/S0140-6736(25)01665-4 40897189

[100] Zannad F, O'Connor CM, Butler J, McMullan CJ, Anstrom KJ, Barash I, et al Vericiguat for patients with heart failure and reduced ejection fraction across the risk spectrum: an individual participant data analysis of the VICTORIA and VICTOR trials. Lancet 2025;406:1351–62. 10.1016/S0140-6736(25)01682-4 40897188

[101] DiFrancesco D . Funny channels in the control of cardiac rhythm and mode of action of selective blockers. Pharmacol Res 2006;53:399–406. 10.1016/j.phrs.2006.03.006 16638640

[102] Savelieva I, Camm AJ. I f inhibition with ivabradine: electrophysiological effects and safety. Drug Saf 2008;31:95–107. 10.2165/00002018-200831020-00001 18217787

[103] Swedberg K, Komajda M, Böhm M, Borer JS, Ford I, Dubost-Brama A, et al Ivabradine and outcomes in chronic heart failure (SHIFT): a randomised placebo-controlled study. Lancet 2010;376:875–85. 10.1016/S0140-6736(10)61198-1 20801500

[104] Böhm M, Borer J, Ford I, Gonzalez-Juanatey JR, Komajda M, Lopez-Sendon J, et al Heart rate at baseline influences the effect of ivabradine on cardiovascular outcomes in chronic heart failure: analysis from the SHIFT study. Clin Res Cardiol 2013;102:11–22. 10.1007/s00392-012-0467-8 22575988

[105] Komajda M, Isnard R, Cohen-Solal A, Metra M, Pieske B, Ponikowski P, et al Effect of ivabradine in patients with heart failure with preserved ejection fraction: the EDIFY randomized placebo-controlled trial. Eur J Heart Fail 2017;19:1495–503. 10.1002/ejhf.876 28462519

[106] Digitalis Investigation Group . The effect of digoxin on mortality and morbidity in patients with heart failure. N Engl J Med 1997;336:525–33. 10.1056/NEJM199702203360801 9036306

[107] Ahmed A, Rich MW, Fleg JL, Zile MR, Young JB, Kitzman DW, et al Effects of digoxin on morbidity and mortality in diastolic heart failure: the ancillary digitalis investigation group trial. Circulation 2006;114:397–403. 10.1161/CIRCULATIONAHA.106.628347 16864724 PMC2628473

[108] Bavendiek U, Großhennig A, Schwab J, Berliner D, Rieth A, Maier LS, et al Digitoxin in patients with heart failure and reduced ejection fraction. N Engl J Med 2025;393:1155–65. 10.1056/NEJMoa2415471 40879434

[109] Sattar N, Lee MMY, Kristensen SL, Branch KRH, Del Prato S, Khurmi NS, et al Cardiovascular, mortality, and kidney outcomes with GLP-1 receptor agonists in patients with type 2 diabetes: a systematic review and meta-analysis of randomised trials. Lancet Diabetes Endocrinol 2021;9:653–62. 10.1016/S2213-8587(21)00203-5 34425083

[110] Ferreira JP, Saraiva F, Sharma A, Vasques-Nóvoa F, Angélico-Gonçalves A, Leite AR, et al Glucagon-like peptide 1 receptor agonists in patients with type 2 diabetes with and without chronic heart failure: a meta-analysis of randomized placebo-controlled outcome trials. Diabetes Obes Metab 2023;25:1495–502. 10.1111/dom.14997 36722252

[111] Kosiborod MN, Abildstrøm SZ, Borlaug BA, Butler J, Rasmussen S, Davies M, et al Semaglutide in patients with heart failure with preserved ejection fraction and obesity. N Engl J Med 2023;389:1069–84. 10.1056/NEJMoa2306963 37622681

[112] Kosiborod MN, Petrie MC, Borlaug BA, Butler J, Davies MJ, Hovingh GK, et al Semaglutide in patients with obesity-related heart failure and type 2 diabetes. N Engl J Med 2024;390:1394–407. 10.1056/NEJMoa2313917 38587233

[113] Butler J, Shah SJ, Petrie MC, Borlaug BA, Abildstrøm SZ, Davies MJ, et al Semaglutide versus placebo in people with obesity-related heart failure with preserved ejection fraction: a pooled analysis of the STEP-HFpEF and STEP-HFpEF DM randomised trials. Lancet 2024;403:1635–48. 10.1016/S0140-6736(24)00469-0 38599221 PMC11317105

[114] Willard FS, Douros JD, Gabe MB, Showalter AD, Wainscott DB, Suter TM, et al Tirzepatide is an imbalanced and biased dual GIP and GLP-1 receptor agonist. JCI Insight 2020;5:e140532. 10.1172/jci.insight.140532 32730231 PMC7526454

[115] Qin W, Yang J, Ni Y, Deng C, Ruan Q, Ruan J, et al Efficacy and safety of once-weekly tirzepatide for weight management compared to placebo: an updated systematic review and meta-analysis including the latest SURMOUNT-2 trial. Endocrine 2024;86:70–84. 10.1007/s12020-024-03896-z 38850440 PMC11445313

[116] Packer M, Zile MR, Kramer CM, DiMaria JM, Baum SJ, Litwin SE, et al Influence of type 2 diabetes on the effects of tirzepatide in patients with heart failure and a preserved ejection fraction with obesity: a prespecified stratification-based analysis. J Am Coll Cardiol 2025;86:696–707. 10.1016/j.jacc.2025.06.058 40903131

[117] Margulies KB, Hernandez AF, Redfield MM, Givertz MM, Oliveira GH, Cole R, et al Effects of liraglutide on clinical stability among patients with advanced heart failure and reduced ejection fraction: a randomized clinical trial. JAMA 2016;316:500–8. 10.1001/jama.2016.10260 27483064 PMC5021525

[118] Neves JS, Vasques-Nóvoa F, Borges-Canha M, Leite AR, Sharma A, Carvalho D, et al Risk of adverse events with liraglutide in heart failure with reduced ejection fraction: a post hoc analysis of the FIGHT trial. Diabetes Obes Metab 2023;25:189–97. 10.1111/dom.14862 36082522 PMC9742170

[119] Jorsal A, Kistorp C, Holmager P, Tougaard RS, Nielsen R, Hänselmann A, et al Effect of liraglutide, a glucagon-like peptide-1 analogue, on left ventricular function in stable chronic heart failure patients with and without diabetes (LIVE)-a multicentre, double-blind, randomised, placebo-controlled trial. Eur J Heart Fail 2017;19:69–77. 10.1002/ejhf.657 27790809

[120] Neves JS, Leite AR, Mentz RJ, Holman RR, Zannad F, Butler J, et al Cardiovascular outcomes with exenatide in type 2 diabetes according to ejection fraction: the EXSCEL trial. Eur J Heart Fail 2025;27:540–51. 10.1002/ejhf.3478 39381950

[121] Lincoff AM, Brown-Frandsen K, Colhoun HM, Deanfield J, Emerson SS, Esbjerg S, et al Semaglutide and cardiovascular outcomes in obesity without diabetes. N Engl J Med 2023;389:2221–32. 10.1056/NEJMoa2307563 37952131

[122] Joseph P, Roy A, Lonn E, Störk S, Floras J, Mielniczuk L, et al Global variations in heart failure etiology, management, and outcomes. JAMA 2023;329:1650–61. 10.1001/jama.2023.5942 37191704 PMC10189564

[123] Chioncel O, Čelutkienė J, Bělohlávek J, Kamzola G, Lainscak M, Merkely B, et al Heart failure care in the Central and Eastern Europe and Baltic region: status, barriers, and routes to improvement. ESC Heart Fail 2024;11:1861–74. 10.1002/ehf2.14687 38520086 PMC11287314

[124] Biegus J, Tymków R, Butler J, Metra M, Chioncel O, Chopra V, et al Attitudes toward using single-pill combination (polypill) therapy in heart failure: patients’ and physicians’ perspectives. ESC Heart Fail 2026;13:xvag044. 10.1093/eschf/xvag044 41711228 PMC13042294

[125] Baudry G, Monzo L, Petrie MC, Girerd N, Piña IL, Mebazaa A, et al Consistency of HFrEF treatment effect in underrepresented groups in randomized clinical trials. NPJ Cardiovasc Health 2024;1:27. 10.1038/s44325-024-00028-4 41776334 PMC12912368

[126] Mebazaa A, Davison B, Chioncel O, Cohen-Solal A, Diaz R, Filippatos G, et al Safety, tolerability and efficacy of up-titration of guideline-directed medical therapies for acute heart failure (STRONG-HF): a multinational, open-label, randomised, trial. Lancet 2022;400:1938–52. 10.1016/S0140-6736(22)02076-1 36356631

[127] Damasceno A, Saidu H, Cotter G, Davison B, Edwards C, Celutkiene J, et al Socio-economic status and the effect of guideline-directed medical therapy in the STRONG-HF study. ESC Heart Fail 2025;12:1594–605. 10.1002/ehf2.15156 39938529 PMC12055368

[128] Cotter G, Deniau B, Davison B, Edwards C, Adamo M, Arrigo M, et al Optimization of evidence-based heart failure medications after an acute heart failure admission: a secondary analysis of the STRONG-HF randomized clinical trial. JAMA Cardiol 2024;9:114–24. 10.1001/jamacardio.2023.4553 38150260 PMC10753435

[129] Heiat A, Gross CP, Krumholz HM. Representation of the elderly, women, and minorities in heart failure clinical trials. Arch Intern Med 2002;162:1682–8. 10.1001/archinte.162.15.1682 12153370

[130] Tahhan AS, Vaduganathan M, Greene SJ, Fonarow GC, Fiuzat M, Jessup M, et al Enrollment of older patients, women, and racial and ethnic minorities in contemporary heart failure clinical trials: a systematic review. JAMA Cardiol 2018;3:1011–9. 10.1001/jamacardio.2018.2559 30140928

[131] Chioncel O, Davison B, Adamo M, Antohi LE, Arrigo M, Barros M, et al Non-cardiac comorbidities and intensive up-titration of oral treatment in patients recently hospitalized for heart failure: insights from the STRONG-HF trial. Eur J Heart Fail 2023;25:1994–2006. 10.1002/ejhf.3039 37728038

[132] Cerlinskaite-Bajore K, Lam CSP, Sliwa K, Adamo M, Ter Maaten JM, Léopold V, et al Sex-specific analysis of the rapid up-titration of guideline-directed medical therapies after a hospitalization for acute heart failure: insights from the STRONG-HF trial. Eur J Heart Fail 2023;25:1156–65. 10.1002/ejhf.2882 37191154

[133] Arrigo M, Biegus J, Asakage A, Mebazaa A, Davison B, Edwards C, et al Safety, tolerability and efficacy of up-titration of guideline-directed medical therapies for acute heart failure in elderly patients: a sub-analysis of the STRONG-HF randomized clinical trial. Eur J Heart Fail 2023;25:1145–55. 10.1002/ejhf.2920 37246591

[134] Dimond MG, Rosner CM, Lee SB, Shakoor U, Samadani T, Batchelor WB, et al Guideline-directed medical therapy implementation during hospitalization for cardiogenic shock. ESC Heart Fail 2025;12:60–70. 10.1002/ehf2.14863 39327768 PMC11769606

[135] Kresoja KP, Adamo M, Rommel K-P, Stolz L, Karam N, Giannini C, et al Guideline-directed medical therapy assessment in heart failure patients undergoing percutaneous mitral valve repair. ESC Heart Fail 2024;11:1802–7. 10.1002/ehf2.14705 38351672 PMC11098622

